# JAK1 inactivation promotes proliferation and migration of endometrial cancer cells via upregulating the hypoxia-inducible factor signaling pathway

**DOI:** 10.1186/s12964-022-00990-5

**Published:** 2022-11-14

**Authors:** Qin Lin, Zheng Chen, Wei Shi, Zeheng Lv, Xiaoping Wan, Kun Gao

**Affiliations:** 1 Shanghai Key Laboratory of Maternal Fetal Medicine, Shanghai Institute of Maternal-Fetal Medicine and Gynecologic Oncology, Shanghai First Maternity and Infant Hospital, Tongji University School of Medicine, Shanghai, 20092 China; 2 Department of Obstetrics and Gynecology, International Peace Maternity and Child Health Hospital, School of Medicine, Shanghai Jiao Tong University, Shanghai, 200030 China; 3 Department of Clinical Laboratory, Shanghai First Maternity and Infant Hospital, Tongji University School of Medicine, Shanghai, 201204 China

**Keywords:** Endometrial cancer, JAK1, HIF-1α, HIF-2α, Proliferation, Migration

## Abstract

**Background:**

Loss-of-function (LOF) mutations of JAK1, a member of the JAK kinase family, were frequently observed in EC, indicating that JAK1 may act as a tumor suppressor, at least in EC. However, the mechanism of JAK1 mediated regulation of tumorigenesis remains poorly understood.

**Methods:**

The genetic alterations of JAK1 in EC using latest sequencing dataset of EC deposited in TCGA database. The RNA-Seq dataset of EC and normal endometrial tissues from TCGA cohort was analyzed. The expression of JAK1 in EC and normal endometrial tissues were investigated using immunohistochemistry. The expression levels of genes in endometrial cancer cells were detected by quantitative reverse transcription-PCR (RT-qPCR) and western blotting. JAK1 protein was efficiently depleted by the two shRNAs. HIF1/2-α protein was efficiently depleted by siRNAs. JAK1 overexpressed EC cells were generated by an expressing plasmid. The proliferation and migration ability of cancer cells were evaluated by CCK8, colony formation assays and transwell assays. The global transcriptomic changes in JAK1-depleted KLE cells were investigated using RNA-Seq. Gene Ontology (GO) Kyoto Encyclopedia of Genes and Genomes (KEGG) pathway enrichment analysis were used to identify the most significant pathways that were altered in JAK1-depleted KLE cells. The physical association between HIF-1/2α and JAK1 using co-immunoprecipitation (co-IP) assays.

**Results:**

In the present study, we found that JAK1 was frequently mutated and downregulated in EC. JAK1 knockdown promotes EC cell proliferation and migration. JAK1 overexpression reduces EC cell proliferation and migration. We examined the transcriptional profiling changes in JAK1-depleted EC cells and unexpectedly found that the hypoxia inducible factor (HIF) pathway was activated. Mechanistically, JAK1 interacts with HIF-1/2α, and reduces HIF1/2-α protein expression under hypoxia. HIF-1/2α knockdown reverses the JAK1 knockdown–induced growth and migration of EC cells under hypoxia. JAK1 knockdown or pharmacological inhibition of JAK1 kinase activity by Ruxolitinib upregulates transcription of HIF target genes under hypoxia. JAK1 overexpression downregulates transcription of HIF target genes under hypoxia.

**Conclusions:**

These findings provide novel insights into the functional link between JAK1 LOF mutations and abnormal HIF pathway activation in EC and suggest that pharmacological inhibition of HIF1/2 represents a promising therapeutic strategy targeting JAK1-mutated ECs.

**Video Abstract**

**Supplementary Information:**

The online version contains supplementary material available at 10.1186/s12964-022-00990-5.

## Background

Endometrial cancer (EC) is one of the most common malignancies affecting the reproductive system. The incidence of endometrial cancer has markedly increased in recent years. In the United States, it is the fourth most common malignancy among women, with an estimated 65,950 new cases and 12,550 deaths in 2022 [1]. There is an urgent need to better understand the molecular mechanisms underlying endometrial cancer progression. A large-scale genome sequencing performed by The Cancer Genome Atlas (TCGA) on primary EC indicated that EC can be categorized into four distinct molecular subtypes: ultramutated/POLE mutant, hypermutated/microsatellite instability (MSI), copy number low/microsatellite stable (MSS), and copy number high. The identification of key oncogenes and tumor suppressors in each EC subtype and the development of targeted therapies for each subtype may revolutionize the treatment paradigm in EC [2].

In recent decades, studies have revealed that Janus kinases (JAKs) play a crucial role in the regulation of essential cellular mechanisms [3–8]. JAK1, a member of the JAKs family kinases, is a critical signaling kinase for receptor cytokines (IL-2, IL-4, IL-7, IL-9, IL-15, and IL-21), pro-inflammatory cytokines including IL-6, and IFN [9]. The JAK1 pathway is involved in many types of human cancers, including hepatocellular carcinoma, gastric cancer, colorectal cancer, oral squamous cell carcinoma, and endometrial cancer [10–14]. JAK1 loss-of-function (LOF) mutations have been found in endometrial cancer and may facilitate immune evasion [15, 16]. However, the functional role of JAK1 in EC has not been fully elucidated.

HIFs (hypoxia-inducible factors) consist of a heterodimeric structure composed of an O2-sensitive α subunit (HIF-1α and HIF-2α) and an O2-insensitive β subunit (HIF-1β) [17]. High expression of HIF-1α and HIF-2α has been observed in many types of human tumors, including endometrial cancer [18–22].

In the present study, a series of functional analyses in EC cell lines and patient samples were performed to investigate the biological significance of JAK1 inactivation in EC. We established a functional role for JAK1 in suppressing the HIF signaling pathway.

## Methods

### TCGA data acquisition and reprocessing

Expression profiles and somatic mutation data of endometrial cancers were obtained using cBioPortal (https://www.cbioportal.org/). Expression profiles of normal endometrial tissues were obtained using the GDC Data Portal (https://portal.gdc.cancer.gov/projects/TCGA-UCEC). Normalization and differential expression analyses of transcriptome data were conducted using the edgeR package.

### Ethics statement and tissue samples collection

This study was approved by the Human Investigation Ethics Committee of Shanghai First Maternity and Infant Hospital, Tong Ji University School of Medicine. EC and normal endometrial tissues were collected after written informed consent from the patients. Paraffin-embedded tissue samples for immunohistochemistry were obtained from patients who underwent surgical treatment from 2019 to 2021 (Additional file 5: Table S1). Two independent pathologists verified the histological diagnosis of all tissue samples. None of the patients had undergone hormone therapy, radiotherapy, or chemotherapy prior to surgery.

### Immunohistochemistry

All tissue sections (4 mm thick) were prepared in paraffin-embedded specimens. Staining was performed using primary antibodies as follows: rabbit polyclonal antibody against JAK1 (1:100; CST, Danvers, MA, USA). Two independent pathologists who were blinded to the clinical and pathological data evaluated the specimens. Sections were evaluated according to semi-quantitative immunoreactivity scores. The percentage of positive staining was scored as follows: 0 = 0–5%, 1 = 6%-25%, 2 = 26–50%, 3 = 51–75% and 4 > 75%. Staining intensity was scored as follows: 0 = none, 1 = weak, 2 = moderate, and 3 = strong. For each specimen, the final score (Immunohistochemistry scores, IS) was the multiply of the values of the two parameters.

### Cell culture and hypoxic conditions

293 T, KLE, SPEC-2, AN3CA, Ishikawa, HEC-1A, and HEC-1B were obtained from the Chinese Academy of Sciences Committee Type Culture Collection cell bank (Shanghai, China). These cell lines were grown in Dulbecco’s modified Eagle’s medium (DMEM)/F12 (Gibco, Auckland, NZ) supplemented with 10% fetal bovine serum (Gibco) in a humidified atmosphere of 5% CO_2_ at 37 °C. Ruxolitinib was prepared for use in cell culture. For hypoxia experiments, cells were grown in an in vitro hypoxic (2% O_2_) container system (BD Diagnostics), and protein and RNA were collected immediately when cells were removed from the hypoxic container [23].

### Transient transfection

shRNAs and siRNAs were purchased from GeneCHEM (Shanghai, China). The sequences are listed in Additional file 5: Tables S2 and S3. The JAK1 expression plasmid, PCDH, was purchased from GenePharma Biotech (Shanghai, China). The Myc-tagged JAK1 and Flag-tagged HIF-1/2α expression plasmids were purchased from WZ Biosciences Inc (Shanghai, China). Transient transfection was performed using LipofectamineTM 2000 (Invitrogen) according to the manufacturer’s protocol.

### RT-qPCR

Total RNA was extracted from cell lines using TRIzol (Invitrogen), and cDNA was prepared using a reverse transcriptase kit (TaKaRa) according to the manufacturer’s instructions. cDNA was analyzed by real-time PCR using SYBR Premix Ex Taq (TaKaRa) in an Eppendorf Mastercycler ep realplex. The housekeeping gene, GAPDH, was used as an internal control. Data were calculated using the 2^−△△Ct^ formula. The primer sequences used are listed in Additional file 5: Table S4. The experiments were repeated at least three times.

### Western blotting

Proteins were extracted using the RIPA kit (Beyotime, Shanghai, China) containing a 1% dilution of the protease inhibitor PMSF (Beyotime). Protein concentrations were determined using a BCA Protein Assay kit (Beyotime). Equal amounts of protein were loaded into each lane of an SDS-PAGE gel for protein separation and transferred to polyvinylidene fluoride (PVDF) membranes (Millipore). Membranes were blocked and then incubated with rabbit polyclonal antibody against JAK1 (1:1000; CST), rabbit polyclonal antibody against HIF-1α (1:1000, CST), rabbit polyclonal antibody against HIF-2α (1:1000, CST), and rabbit polyclonal antibody against GAPDH (1:5000, CST) individually at 4℃ overnight. HRP-conjugated Goat anti-rabbit antibodies (1:2000, Proteintech) were used to detect the bound primary antibodies.

### RNA-sequencing and data analysis

RNA extracted from KLE cells and KLE cells transfected with JAK1 shRNA was used for RNA-sequencing (APExBIO). Differentially expressed genes ((DEGs) between KLE cells and KLE cells transfected with JAK1 shRNA were determined using DESeq2 and the Wald hypothesis test. Genes were considered significant and reported according to the following criteria: FDR < 0.05 and |log2FoldChange|> 1. Heat maps and hierarchical clustering were performed with R software.

To further explore the potential functions of JAK1 in the pathology of EC, DEGs were analyzed using Gene Ontology (GO, http://www.geneontology.org/) and the Kyoto Gene and Genomic Encyclopedia (KEGG, https://www.kegg.jp/) pathway enrichment analysis. Differences were considered statistically significant at *P* < 0.05.

### Cell proliferation

For Cell proliferation assays, cells were seeded into 96-well plates at 1 × 10^3^ cells/well and cultured for 1–5 days. Cell proliferation assays were performed using the CCK-8 Kit (Dojindo). Absorbance was measured at 450 nm using a Multimode Plate Reader (Molecular Devices, USA).

### Colony formation assays

For colony formation assays, cells were seeded into 6-well plates. When identifiable cell clones had formed, the colonies were fixed with methanol and stained with 0.5% crystal violet. All experiments were repeated at least three times.

### Cell migration assays

Cells were suspended in 200 μl serum-free medium and plated at a density of 6 × 10^4^ cells/well in 6.5 mm transwell chambers equipped with 8.0 μm pore-size polycarbonate membranes. The complete medium (600 μl) was added to the lower chamber. After incubation for 48 h, cells were fixed in 4% paraformaldehyde and stained with crystal violet. The cells that migrated to the basal side of the membrane were counted using a microscope.

### Co-immunoprecipitation assay (Co-IP)

Co-IP assays were performed using Pierce Classic Magnetic IP/Co-IP Kit (Thermo Fisher) and Flag Tag IP/Co-IP Kit (Biolinkedin, China) according to the manufacturer’s instructions. Co-immunoprecipitated proteins were analyzed by western blotting as described above. Anti-Flag and anti-Myc antibodies were purchased from Sigma-Aldrich.

### Statistical analysis

All data were statistically analyzed using Graphpad prism 7.0. Measured data were assessed using unpaired Student’s t-test or one-way ANOVA for multiple comparisons, and the χ^2^ test for 2 × 2 tables was used to compare the categorical data. *p < 0.05 **p < 0.01, ***p < 0.001, ****p < 0.0001.

## Results

### JAK1 was frequently mutated and downregulated in EC

**Fig. 1:**
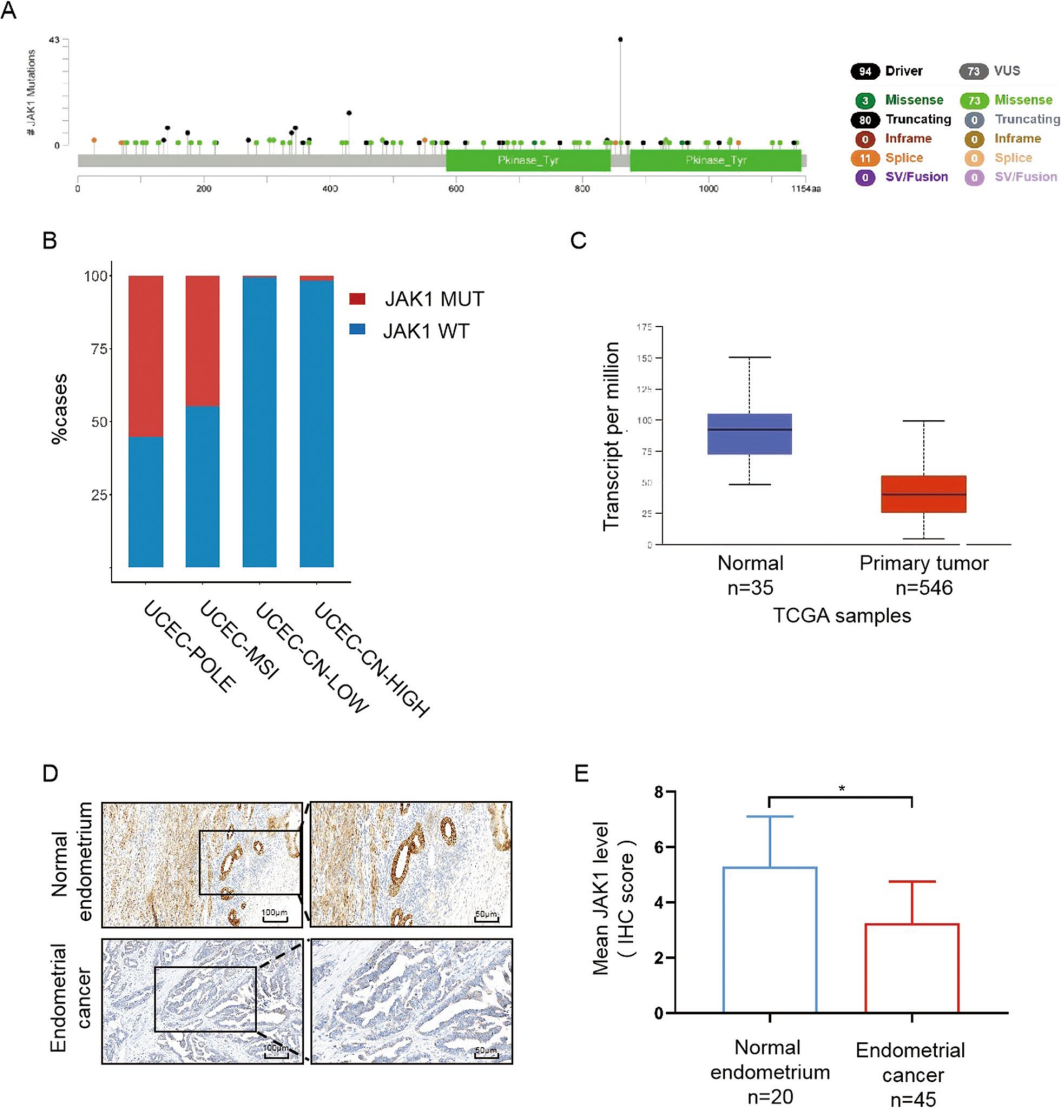
JAK1 is frequently mutated and downregulated in EC. **A** Schematics of the JAK1 proteins show the positions of individual somatic mutations identified in the endometrial cancer TCGA cohort. **B** JAK1 mutations were more frequently detected in the microsatellite instability (MSI) subgroup and POLE-mutated subgroup. **C** The relative mRNA expression of JAK1 in normal endometrial tissues and endometrial cancer tissues from TCGA cohort. **D** Representative images of JAK1 IHC in normal and EC specimens. Original magnification 200×, scale bar, 100 μm (left); 400×, scale bar, 50 μm (right). **E** Immunohistochemistry scores (IS) of JAK1 in normal endometrium (n = 20) and EC tissues (n = 45)

To evaluate the genetic alterations of JAK1 in EC, we examined the latest sequencing dataset of EC deposited in TCGA database. A total of 546 cases matched with whole-exome sequencing (WXS) and RNA-Seq data were included for further analysis. A total of 167 JAK1 mutations occurred in approximately 30.6% (167/546) of the tumor specimens, including 80 truncating, 76 missense, and 11 splice site mutations (Fig. 1A). The high incidence of truncating mutations (80/167)strongly indicated that JAK1 is frequently inactivated in EC. Moreover, JAK1 mutations were detected more frequently in the MSI and POLE-mutated subgroups than in the low/high copy number groups (Fig. 1B). We then analyzed the RNA-Seq dataset of EC and normal endometrial tissues from TCGA cohort. JAK1 transcript levels were markedly lower in EC tissues than in normal endometrial tissues (*p* < 0.001) (Fig. 1C). Immunohistochemical analysis was performed to detect JAK1 protein levels in 45 EC tissues and 20 normal endometrial tissues, and the results showed that JAK1 protein was markedly downregulated in EC tissues compared to normal endometrial tissues (Fig. 1D, E). Taken together, these results indicate that JAK1 is frequently mutated and that JAK1 protein is downregulated in ECs.

### JAK1 suppresses EC cell growth and migration

**Fig. 2:**
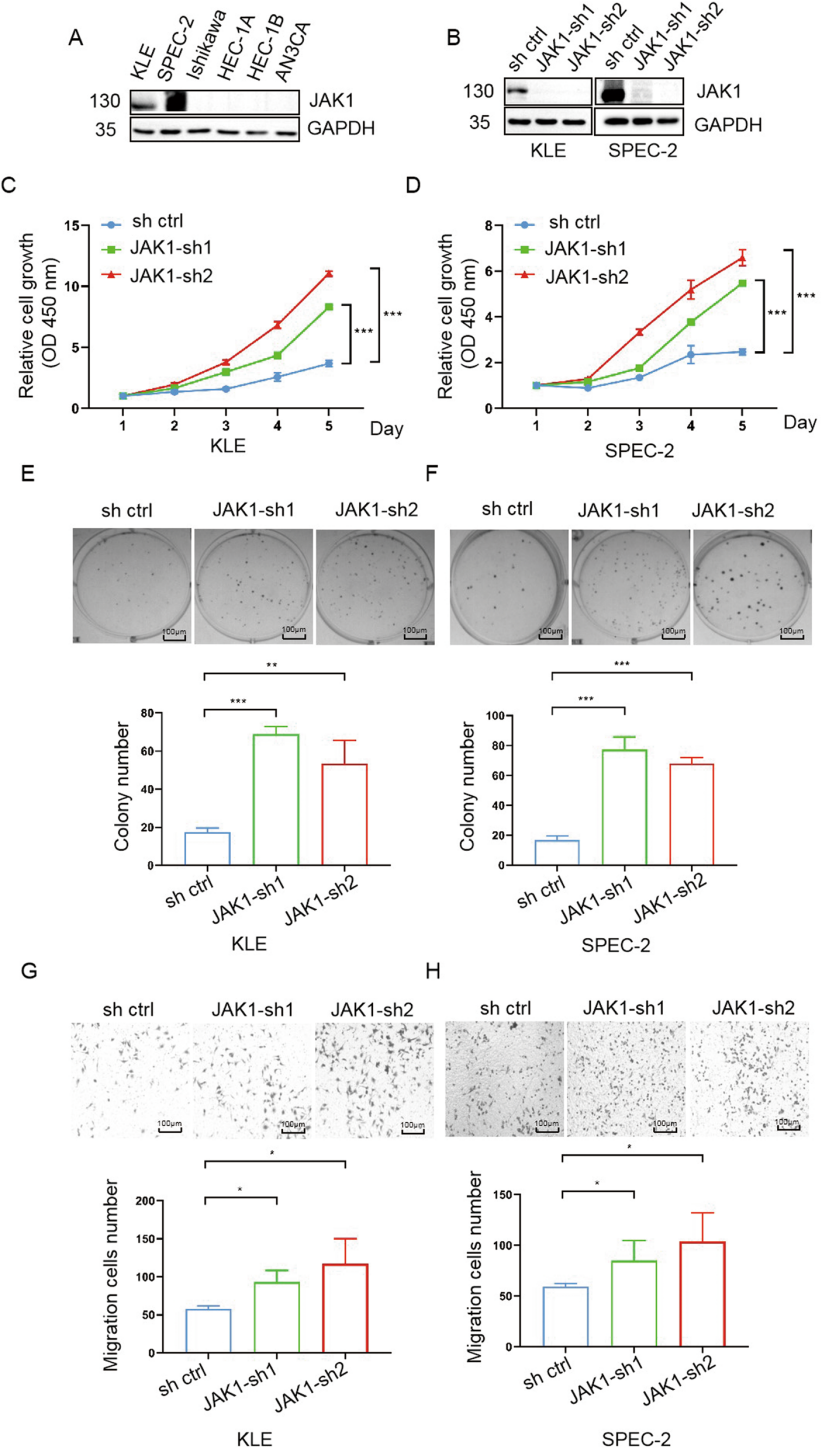
JAK1 suppresses EC cell growth and migration. **A** Western blotting of the indicated proteins in WCLs (whole cell lysates) from six endometrial cancer lines. **B** Western blotting of the indicated proteins in WCLs from KLE and SPEC-2 cells expressing sh ctrl, JAK1-sh1, or JAK1-sh2. **C**, **D** CCK-8 cell proliferation analysis of KLE and SPEC-2 cells expressing sh ctrl, JAK1-sh1, or JAK1-sh2. Data are shown as the mean ± SD (n = 3). **E**, **F** Colony formation analysis of KLE and SPEC-2 cells expressing sh ctrl, JAK1-sh1, or JAK1-sh2. Data are shown as the mean ± SD (n = 3). Scale bar, 100 μm. **G**, **H** Transwell migration analysis of KLE and SPEC-2 cells expressing sh ctrl, JAK1-sh1, or JAK1-sh2. Data are shown as the mean ± SD (n = 3). Scale bar, 100 μm

Next, we detected JAK1 protein expression in six EC cell lines. JAK1 protein was abundantly expressed in KLE and SPEC-2 cells but was nearly undetectable in Ishikawa, HEC1-A, HEC1-B, and ANC3A cells (Fig. 2A). Therefore, KLE and SPEC-2 cells were selected for JAK1 knockdown and cellular phenotype analyses. JAK1 protein was efficiently depleted by the two shRNAs in KLE and SPEC-2 cells. (Fig. 2B). The effect of JAK1 on EC cell proliferation was assessed using the CCK-8 and colony formation assays. The results of both assays indicated that JAK1 depletion led to a marked increase in cell proliferation compared to control cells (Fig. 2C–F). Transwell assays revealed that JAK1 depletion markedly increased KLE and SPEC-2 cell migration (Fig. 2G, H). Given that JAK1 expression was barely detectable in Ishikawa and HEC1-B cells, we generated cell lines expressing JAK1 or an empty control (Additional file 2: Fig. S1A). We found that JAK1 overexpression markedly decreased EC cell proliferation, as assessed by the CCK-8 and colony formation assays (Additional file 2: Fig. S1B–E). Transwell assays also revealed that JAK1 overexpression markedly decreased Ishikawa and HEC1-B cell migration. Taken together, these results indicate that JAK1 acts as a tumor suppressor to negatively regulate EC cell growth and migration.

### Identification of JAK1 as potential regulator of the HIF pathway

**Fig. 3:**
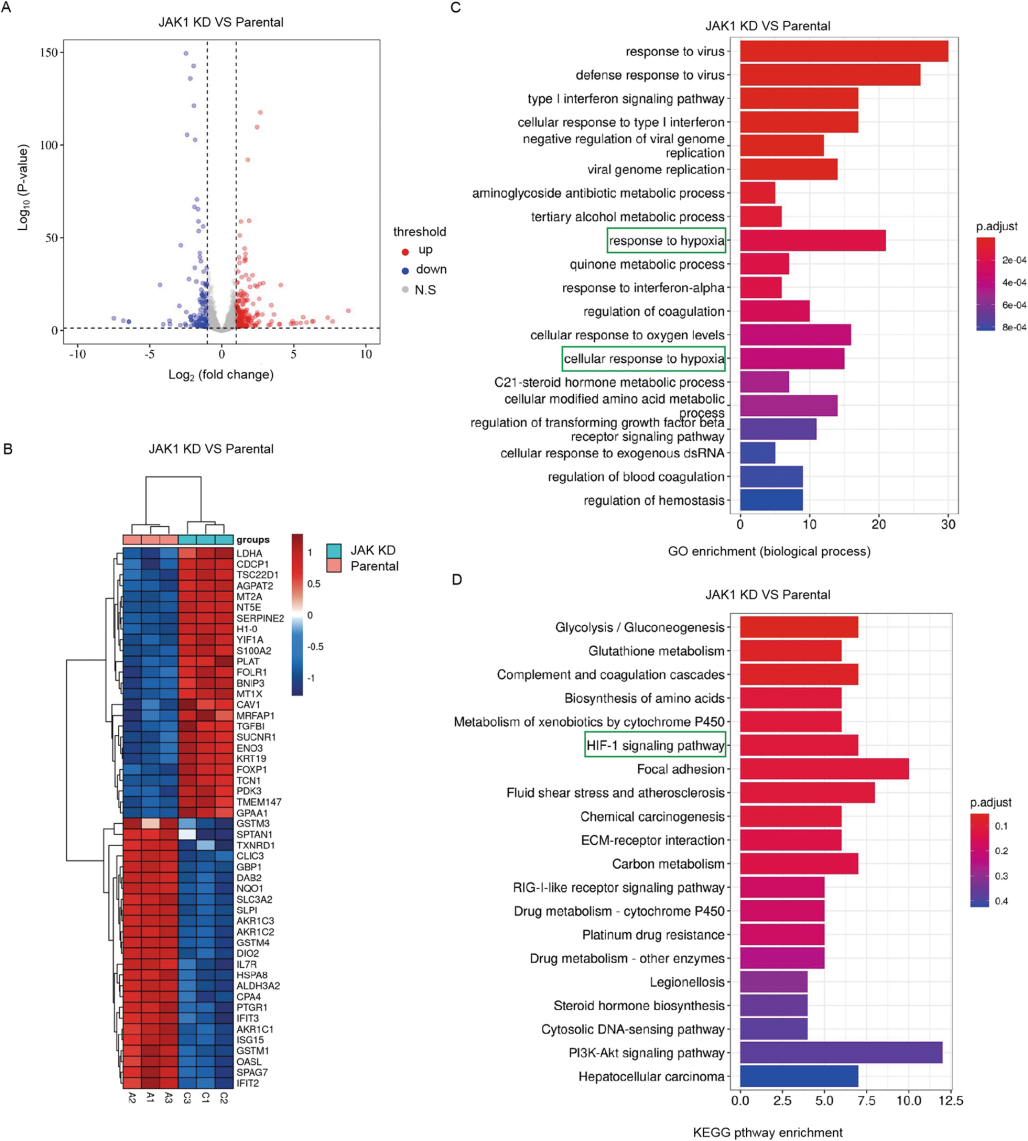
Functional enrichment analyses of the differentially expressed genes by JAK depletion. **A** Volcano plot of the differentially expressed genes in parental and JAK1-knockdown KLE cells. Red dots represent up-regulated genes and blue dots represent down-regulated genes. **B** Heatmap depicting the expression of the top 50 differentially expressed genes in parental and JAK1-knockdown KLE cells. The left vertical axis shows clusters of differentially expressed genes and the right vertical axis represents gene names. Red represents upregulated genes and blue represents downregulated genes. **C** Go analysis of the differentially expressed genes in parental and JAK1-knockdown KLE cells (biological process). **D** KEGG pathway analysis of the differentially expressed genes in parental and JAK1-knockdown KLE cells

To determine the downstream signaling pathways affected by JAK1 depletion in EC cells, we investigated the global transcriptomic changes in JAK1-depleted KLE cells using RNA-Seq. A total of 403 protein-coding genes showed altered expression in JAK1-depleted KLE cells compared to that in control cells (Fig. 3A). A total of 219 genes were upregulated (> twofold), whereas 184 genes were downregulated (< twofold) (Additional file 5: Table S5). The top 50 differentially expressed genes are shown in the heatmap (Fig. 3B). To investigate the functional association of the differentially expressed genes, we conducted Gene Ontology (GO) Kyoto Encyclopedia of Genes and Genomes (KEGG) pathway enrichment analysis to identify the most significant pathways that were altered in JAK1-depleted KLE cells. The results showed that multiple cellular processes, such as ‘response to virus’, ‘response to type I interferon’, ‘virus genome replication’ were significantly altered (Fig. 3C, D). It is not surprising since the JAK-STAT pathway has been reported to play fundamental roles in these processes [24, 25]. Unexpectedly, the GO and KEGG analyses both indicated that the differentially expressed genes were also enriched in the processes related to hypoxia, such as’response to hypoxia’, ‘cellular response to oxygen level’, and the ‘HIF signaling pathway’ (Fig. 3C, D). The HIF signaling pathway governs multiple key biological processes, such as proliferation, metabolism, and metastasis, in a variety of cancer types, including EC. To the best of our knowledge, no studies have reported that JAK1 has any impact on the HIF signaling pathway in EC. Several well-known transcriptional targets of HIF1/2α, including PDK3 and BNIP3, were among the top upregulated genes affected by JAK1 depletion (Fig. 3B). Taken together, these results indicate that JAK1 may act as a negative regulator of the HIF signaling pathway.

### JAK1 interacts with HIF-1/2α and reduces HIF-1/2α protein levels

**Fig. 4:**
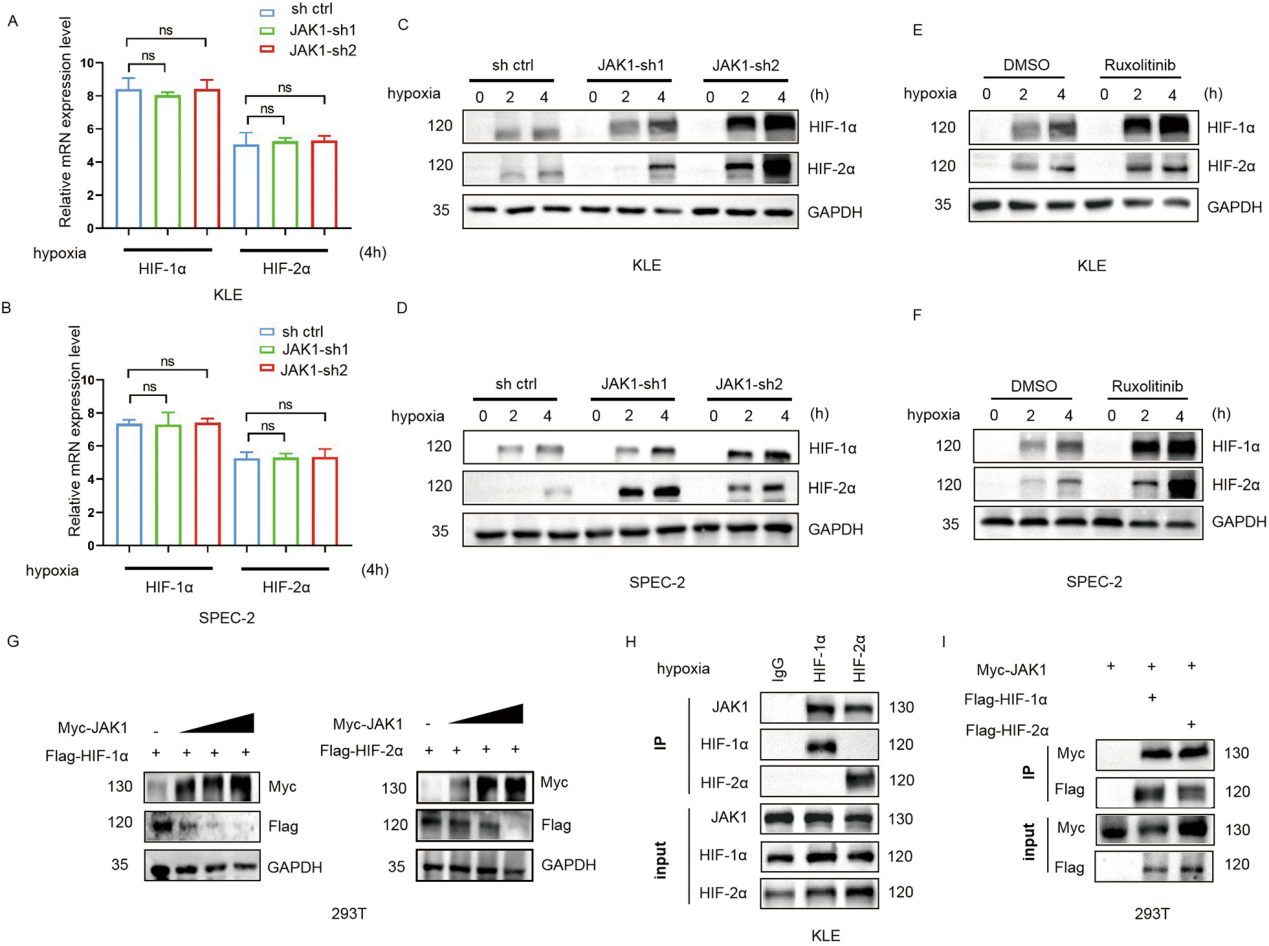
JAK1 interacts with HIF-1/2α and reduces HIF-1/2α protein levels. **A**, **B** RT-qPCR of the indicated mRNAs in WCLs from parental and JAK1-knockdown cells (KLE and SPEC-2 cells). All the cells were incubated under hypoxia (2% O_2_) for 4 h before harvesting. Data are shown as means ± SD (n = 3). **C**, **D** Western blotting of the indicated proteins in WCLs from parental and JAK1-knockdown cells (KLE and SPEC-2 cells). The cells were incubated under hypoxia (2% O_2_). After hypoxia for 0, 2 h, 4 h, cells were harvested, respectively. **E**, **F** Western blotting of the indicated proteins. The cells (KLE and SPEC-2 cells) were treated with DMSO or Ruxolitinib (1 μmol) for 48 h. Then cells were incubated under hypoxia (2% O_2_). After hypoxia for 0, 2 h, 4 h, cells were harvested, respectively. **G** Western blotting of the indicated proteins in WCLs from 293 T cells co-transfected with Flag-tagged HIF-1/2α expression plasmid and different concentrations of Myc-tagged JAK1 expression plasmid (200 ng, 400 ng, 800 ng, 1200 ng). **H** Co-IP assays in KLE cells incubated under hypoxia (2% O_2_) for 24 h. **I**. Co-IP assays in 293 T cells transfected with the indicated plasmids

Given that the aforementioned results suggested that JAK1 depletion upregulates HIF transcriptional targets, we investigated whether JAK1 had any impact on the mRNA and protein levels of HIF1α and HIF2α. We found that few changes were observed on the expression level of HIF1α and HIF2α mRNA between parental and JAK1-knockdown cells (KLE and SPEC-2 cells) under hypoxia (Fig. 4A and B). Protein levels HIF-1α and HIF2α are rapidly stabilized under hypoxia but degraded under normoxia. As expected, hypoxic treatment (0.2% oxygen) led to a dramatic increase in the protein levels of HIF1α and HIF2α in a time-dependent manner. Notably, hypoxia-induced HIF-1/2α protein expression was more abundant in JAK1-depleted KLE and SPEC-2 cells than in control KLE and SPEC-2 cells (Fig. 4C and D). JAK1 is a protein kinase; thus, we assessed whether HIF-1/2α protein levels were altered when the kinase activity of JAK1 was inhibited. We found that HIF-1/2α protein was increased in KLE and SPEC-2 cells treated with Ruxolitinib, a potent JAK1/2 inhibitor (Fig. 4E and F). We also found that exogenously overexpressing Myc-JAK1 reduced Flag-HIF-1/2α protein levels in a dose-dependent manner (Fig. 4G). We detected a physical association between HIF-1/2α and JAK1 at endogenous levels in KLE cells under hypoxia using a co-immunoprecipitation assay (Fig. 4H). Finally, we demonstrated that exogenously overexpressing Myc-JAK1 interacted with Flag-HIF-1/2α (Fig. 4I). Taken together, these results indicated that JAK1 may reduce HIF-1/2α protein expression by directly interacting with HIF-1/2α.

### HIF-1/2α knockdown reverses the JAK1 knockdown–induced growth and migration of EC cells

**Fig. 5:**
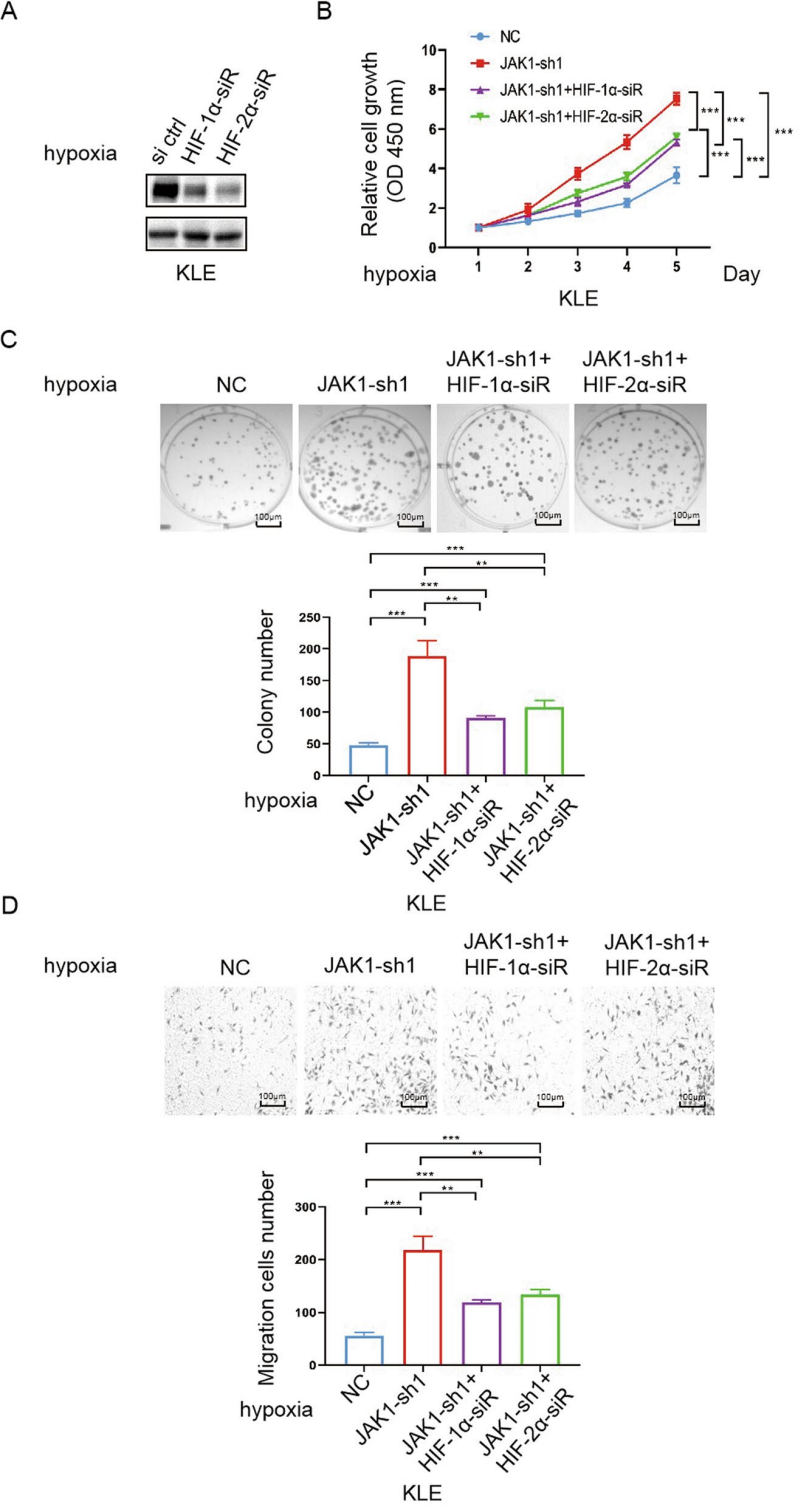
HIF-1/2α knockdown reverses the JAK1 knockdown–induced growth and migration of EC cells. **A** Western blotting of the indicated proteins in WCLs from KLE cells expressing si ctrl, HIF-1α-siR, or HIF-2α-siR. The cells were incubated under hypoxia (2% O_2_). **B** CCK-8 cell proliferation analysis of KLE cells expressing NC (negative control), JAK1-sh1, JAK1-sh1 + HIF-1α-siR, or JAK1-sh1 + HIF-2α-siR. The cells were incubated under hypoxia (2% O_2_). Data are shown as the mean ± SD (n = 3). **C** Colony formation analysis of KLE cells expressing NC (negative control), JAK1-sh1, JAK1-sh1 + HIF-1α-siR, or JAK1-sh1 + HIF-2α-siR. The cells were incubated under hypoxia (2% O_2_). Data are shown as the mean ± SD (n = 3). Scale bar, 100 μm. **D** Transwell migration analysis of IKLE cells expressing NC (negative control), JAK1-sh1, JAK1-sh1 + HIF-1α-siR, or JAK1-sh1 + HIF-2α-siR. The cells were incubated under hypoxia (2% O_2_). Data are shown as the mean ± SD (n = 3). Scale bar, 100 μm

To investigate the possibility that the involvement of HIF-1/2α in the cell phenotype changes caused by JAK1 knockdown, HIF-1/2α were knockdown by siRNA (Fig. 5A). We found that knockdown of HIF-1/2α in JAK1 knockdown KLE cells reversed JAK1 knockdown–induced increase in vitro cell growth and migration (Fig. 5B–D).

### JAK1 downregulated the transcription of the HIF downstream target genes

**Fig. 6:**
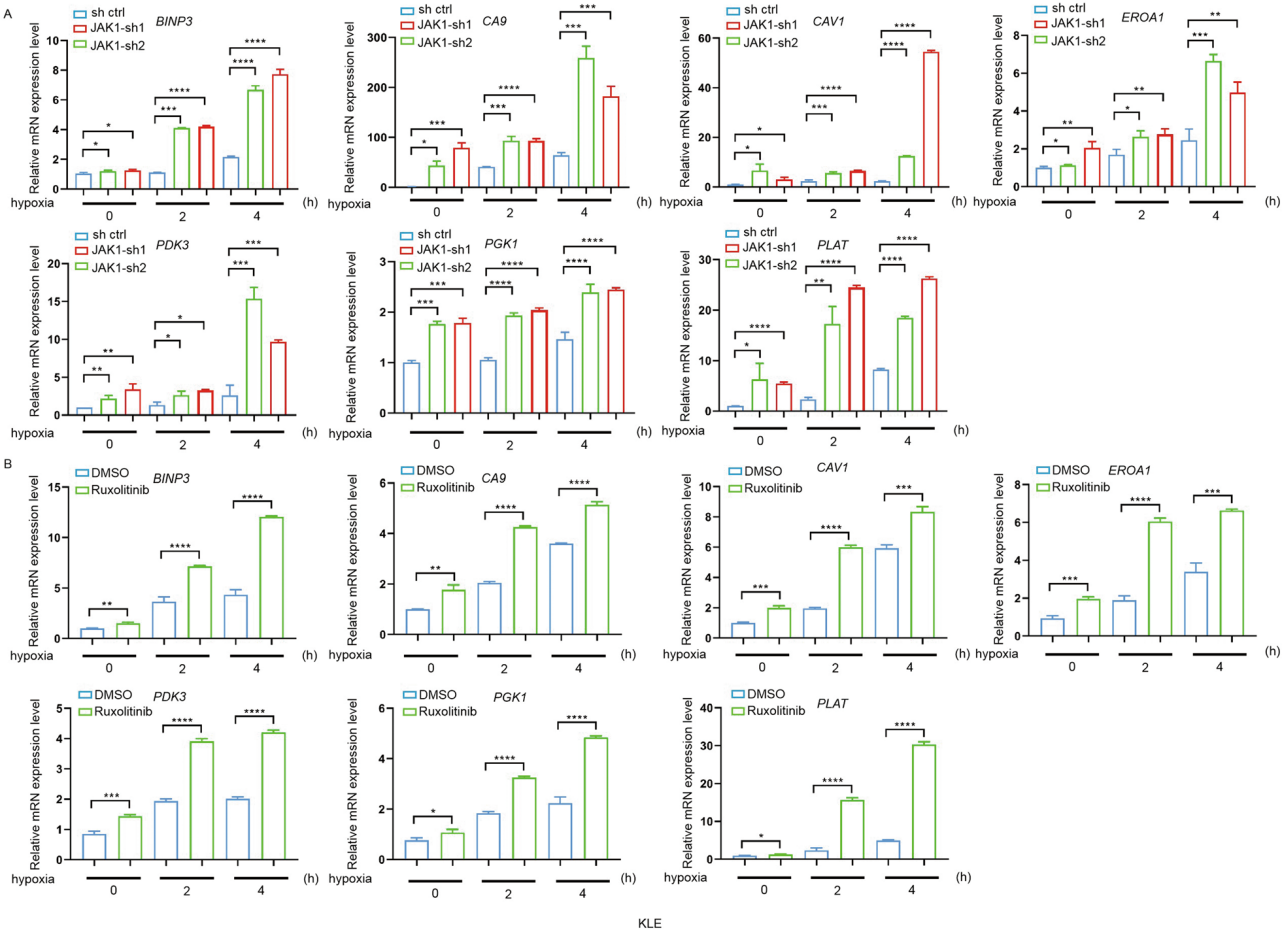
JAK1 knockdown elevates the expression of HIF downstream genes in KLE cells. **A** RT-qPCR measurement of the mRNA expression of HIF downstream genes in parental and JAK1-knockdown KLE cells. The cells were incubated under hypoxia (2% O_2_). After hypoxia for 0, 2 h, 4 h, cells were harvested, respectively. Data are shown as the mean ± SD (n = 3). **B** RT-qPCR measurement of the mRNA expression of HIF downstream genes in different KLE cells. The cells were treated with DMSO or Ruxolitinib (1 μmol) for 48 h. Then cells were incubated under hypoxia (2% O_2_). After hypoxia for 0, 2 h, 4 h, cells were harvested, respectively. Data are shown as the mean ± SD (n = 3)

To further validate whether JAK1 affects the transcriptional outputs of the HIF signaling pathway, the mRNA expression of HIF downstream target genes was assessed. We found that hypoxia-induced upregulation of HIF downstream target genes, including BNIP3, CA9, CAV1, PDK3, PGK1, PLAT, and EROA1, was potentiated in JAK1-depleted KLE and SPEC-2 cells (Fig. 6A and Additional file 3: Fig. S2A). Similar results were observed in Ruxolitinib-treated KLE and SPEC-2 cells (Fig. 6B and Additional file 3: Fig. S2B). JAK1 overexpression downregulated transcription of HIF target genes under hypoxia in Ishikawa cells (Additional file 4: Fig. S3). Taken together, these results indicate that JAK1 negatively modulates the transcriptional outputs of the HIF signaling pathway in a kinase activity-dependent manner.

## Discussion

In the present study, we demonstrated that knockdown or overexpression of JAK1 in endometrial cancer cell lines leads to changes in EC cell growth and migration potential in vitro. However, the underlying molecular mechanisms were explored, and we propose that JAK1 may promote EC tumorigenesis and progression via the HIF pathway.

Hypoxia is recognized as one of the intrinsic features of solid tumors and is associated with aggressive phenotypes, including resistance to radiation and chemotherapy, metastasis, and poor patient prognosis [26, 27]. Adaptation to hypoxia is primarily mediated through the activation of a family of transcription factors. HIFs, as a means of an adaptive response to a hypoxic environment, are intrinsic markers of tumor hypoxia. HIF-1α is predominantly involved in the early stages of cancer, whereas HIF-2α is actively involved in the later stages [28].

HIFs play a critical role in the adaptation of tumor cells to hypoxia by upregulating plenty of transcription targets. It is well known that HIFs become stabilized and activated at low oxygen levels, whereas normoxia leads to quick degradation of the HIF-1/2α. Our results showed that JAK1 directly interacts with HIF-1/2α, and inhibition of JAK1 kinase activity elevated HIF-1/2α protein levels. JAK1 may directly phosphorylate HIF-1/2α to facilitate HIF-1/2α degradation by CRL2-VHL ubiquitin E3 ligase. Alternatively, JAK1 may phosphorylate other substrates, which may indirectly regulate HIF-1/2α protein stability. Therefore, further studies are required to identify the detailed mechanism involved in regulation of JAK1 on HIF-1/2α.

## Conclusions

**Fig. 7:**
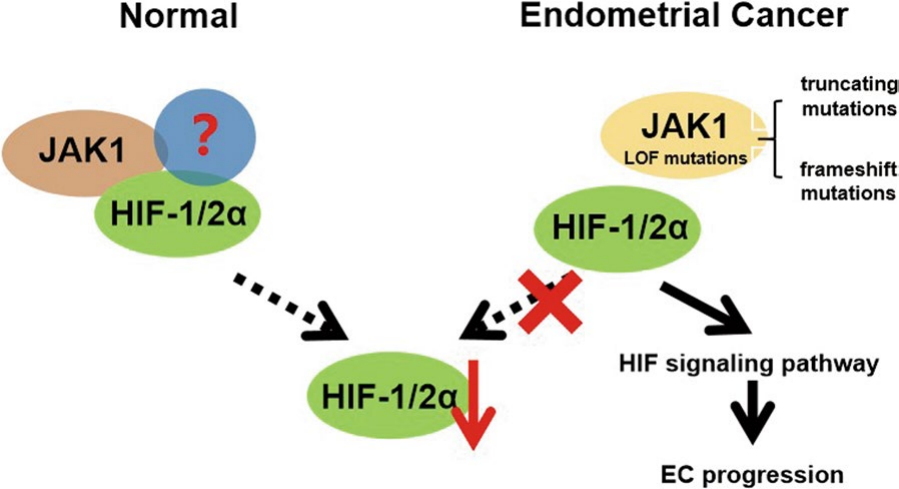
Schematic diagram of the JAK1-HIF-1/2α pathway in EC. Left: In normal cells, JAK1 downregulates the expression of HIF-1/2α. Right: In EC cells, JAK1 LOF mutations promote EC tumorigenesis via activating HIF signaling pathway

In certain tumors, JAK dysfunction has been attributed to loss- or gain-of-function mutations that drive tumor growth and metastasis [29]. JAK1 LOF mutations, including truncating and frameshift mutations, facilitate tumorigenesis via tumor immune evasion in EC [15, 16, 30]. Therefore, our study revealed a new possible molecular pathway by which JAK1 inactivation in EC cells could promote EC tumorigenesis and progression by activating the HIF signaling pathway (Fig. 7). Elucidation of JAK1 mutation-driven EC tumorigenesis is helpful in designing HIF-targeting therapies for JAK1-mutated ECs.

## Supplementary Information

**Additional file 2**: **Fig. S1**. JAK1 overexpression suppresses EC cell growth and migration.

**Additional file 3**: **Fig. S2**. JAK1 knockdown elevates the expression of HIF downstream genes in SPEC-2 cells.

**Additional file 4**: **Fig. S3**. JAK1 overexpression downregulates the expression of HIF downstream genes in Ishikawa cells.

**Additional file 5**. Supplementary Tables.

## Data Availability

All data generated or analyzed during this study are included in this published article.

## Abbreviations

JAK: Janus kinases
EC: Endometrial cancer
LOF: Loss-of-function
HIF: Hypoxia inducible factor
TCGA: The Cancer Genome Atlas
GO: Gene Ontology
KEGG: Kyoto Encyclopedia of Genes and Genomes
JAK1-sh1/2: ShRNA against JAK1
EV/sh ctrl: Control vector
