# Supplementary figures and images for: JAK1 inactivation promotes proliferation and migration of endometrial cancer cells via upregulating the hypoxia-inducible factor signaling pathway
Source: Cell Commun Signal. 2022 Nov 14;20:177. doi: 10.1186/s12964-022-00990-5 (PMC9661757; doi:10.1186/s12964-022-00990-5)

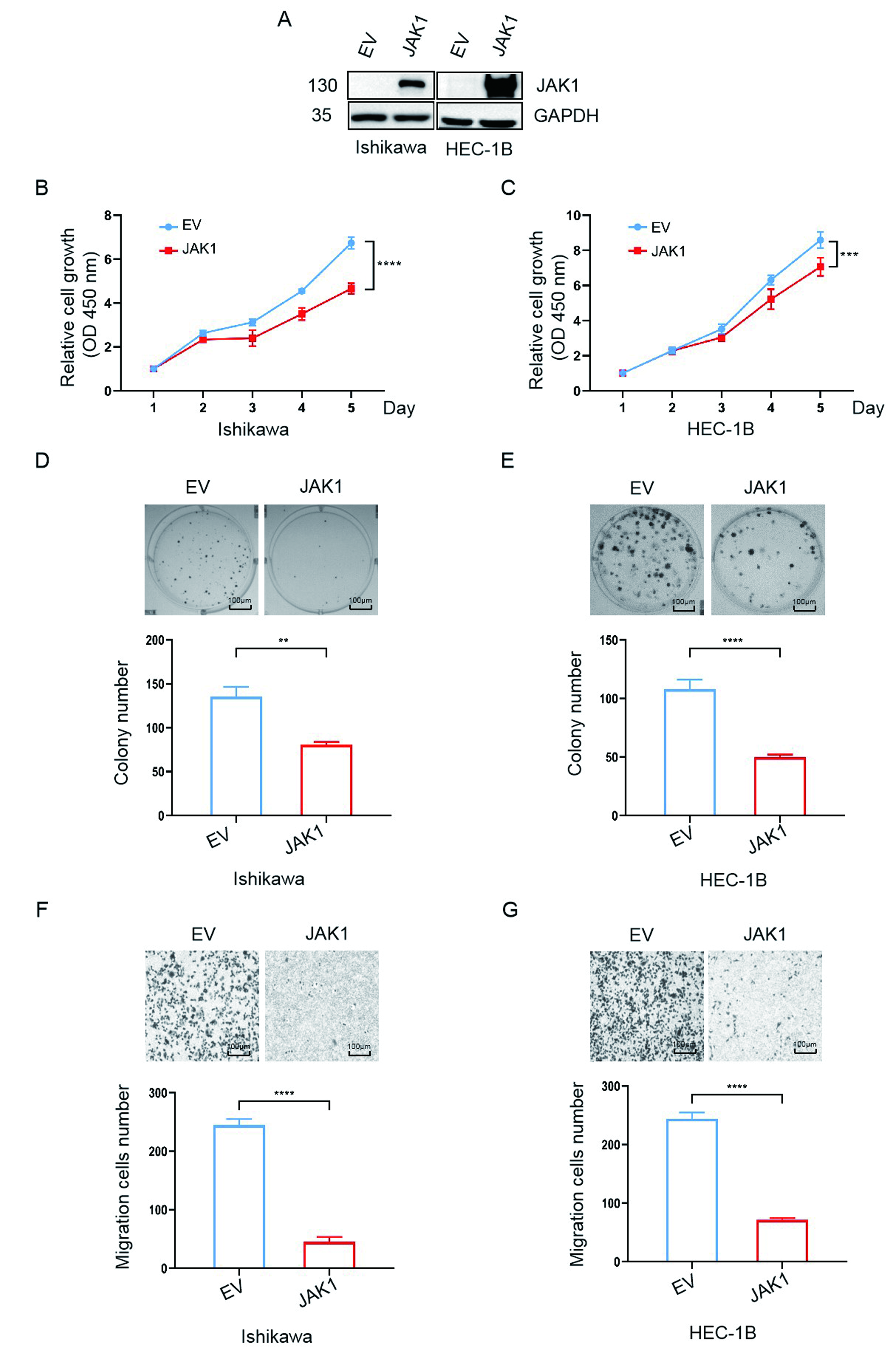

Supplement: Supplementary file 2 — Additional file 2: Fig. S1. JAK1 overexpression suppresses EC cell growth and migration. [file 12964_2022_990_MOESM2_ESM.tif]

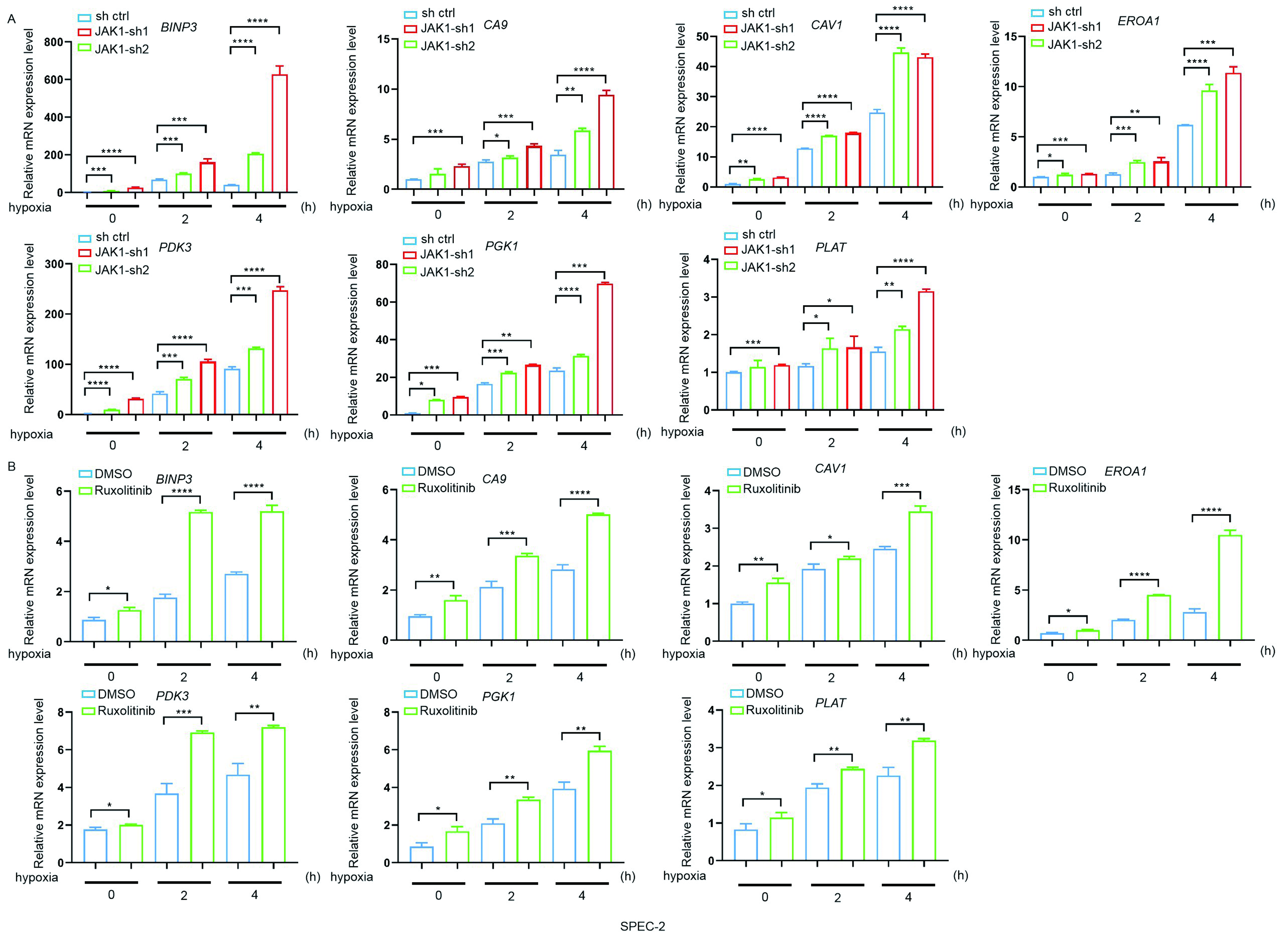

Supplement: Supplementary file 3 — Additional file 3: Fig. S2. JAK1 knockdown elevates the expression of HIF downstream genes in SPEC-2 cells. [file 12964_2022_990_MOESM3_ESM.tif]

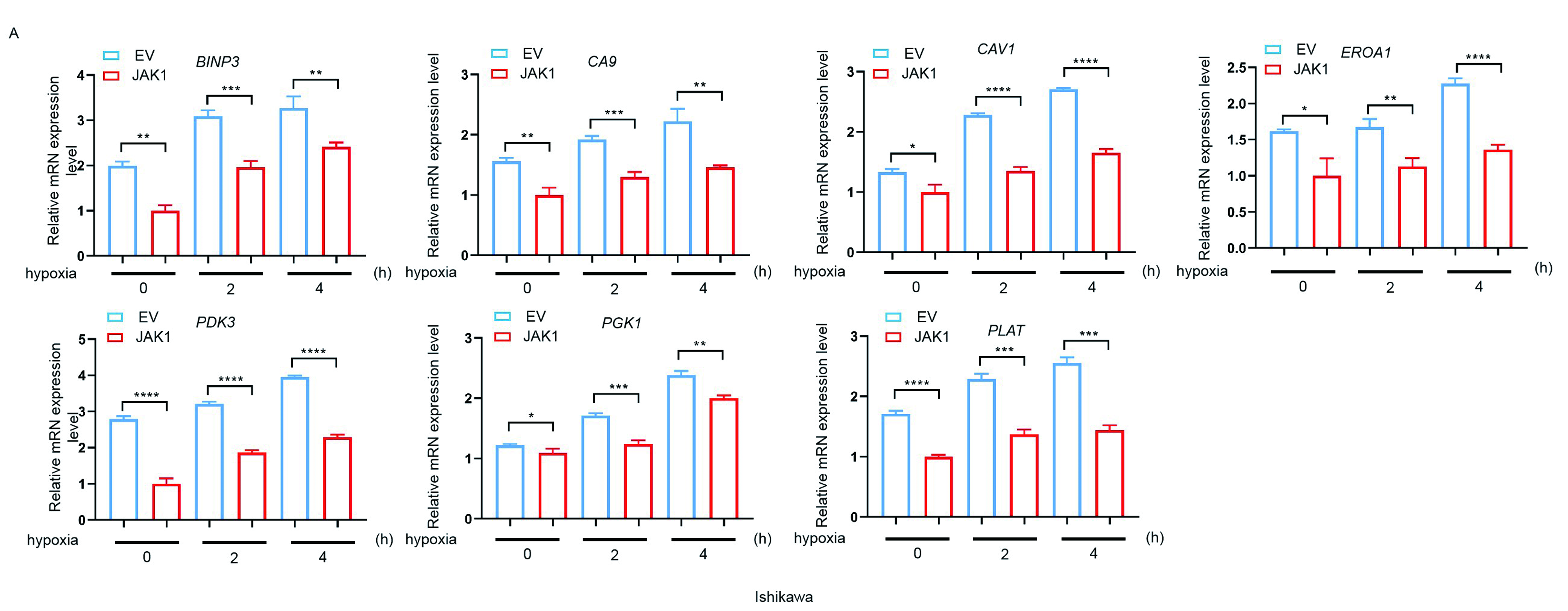

Supplement: Supplementary file 4 — Additional file 4: Fig. S3. JAK1 overexpression downregulates the expression of HIF downstream genes in Ishikawa cells. [file 12964_2022_990_MOESM4_ESM.tif]
